# The mitochondrial C16069T polymorphism, not mitochondrial D310 (D-loop) mononucleotide sequence variations, is associated with bladder cancer

**DOI:** 10.1186/1475-2867-13-120

**Published:** 2013-12-05

**Authors:** Nasser Shakhssalim, Massoud Houshmand, Behnam Kamalidehghan, Abolfazl Faraji, Reza Sarhangnejad, Sepideh Dadgar, Maryam Mobaraki, Rozita Rosli, Mohammad Hossein Sanati

**Affiliations:** 1 Urology and Nephrology Research Center (UNRC), Shahid Labbafinejad Medical Center, Shahid Beheshti University of Medical Sciences, Tehran, Iran; 2 National Institute for Genetic Engineering and Biotechnology, Tehran, Iran; 3 Department of Pharmacy, Faculty of Medicine, University of Malaya (UM), Kuala Lumpur 50603, Malaysia; 4 Medical Genetics Department, Special Medical Center, Tehran, Iran; 5 UPM-MAKNA Cancer Research Laboratory, Institute of Bioscience, Universiti Putra Malaysia, UPM Serdang, Selangor 43400, Malaysia

**Keywords:** Mitochondrial DNA displacement loop, 16069 D-Loop mutation, Urinary bladder neoplasm

## Abstract

**Background:**

Bladder cancer is a relatively common and potentially life-threatening neoplasm that ranks ninth in terms of worldwide cancer incidence. The aim of this study was to determine deletions and sequence variations in the mitochondrial displacement loop (D-loop) region from the blood specimens and tumoral tissues of patients with bladder cancer, compared to adjacent non-tumoral tissues.

**Methods:**

The DNA from blood, tumoral tissues and adjacent non-tumoral tissues of twenty-six patients with bladder cancer and DNA from blood of 504 healthy controls from different ethnicities were investigated to determine sequence variation in the mitochondrial D-loop region using multiplex polymerase chain reaction (PCR), DNA sequencing and southern blotting analysis.

**Results:**

From a total of 110 variations, 48 were reported as new mutations. No deletions were detected in tumoral tissues, adjacent non-tumoral tissues and blood samples from patients. Although the polymorphisms at loci 16189, 16261 and 16311 were not significantly correlated with bladder cancer, the C16069T variation was significantly present in patient samples compared to control samples (p < 0.05). Interestingly, there was no significant difference (p > 0.05) of C variations, including C7TC6, C8TC6, C9TC6 and C10TC6, in D310 mitochondrial DNA between patients and control samples.

**Conclusion:**

Our study suggests that 16069 mitochondrial DNA D-Loop mutations may play a significant role in the etiology of bladder cancer and facilitate the definition of carcinogenesis-related mutations in human cancer.

## Introduction

Human mitochondrial DNA (mtDNA) is a 16569-bp closed circular, double-stranded molecule approximately 1000 copies per cell. mtDNA contains 37 genes, including 13 subunits involved in the electron transport chain, 22 tRNAs, the 12S and 16S rRNAs, and a non-coding region (D-loop) located at nucleotide position 16024–576 (MITOMAP, 2011) [1]. The D-loop region regulates the replication and transcription of mtDNA, where mutations in this region might lead to copy number and/or change in mtDNA gene expression [2].

Bladder cancer is the ninth most common cancer worldwide [3]. According to the latest American Cancer Society statistics, bladder cancer accounts for 7% of all cancers and 3% of all cancer deaths [4,5]. In Iran, bladder cancer accounts for 7.04% of all cancers [6].

Many attempts have been made to develop an urothelial cancer biomarker test to complement or replace urine cytology, including NMP22, BTA stat, BTA TRAK, and FISH. Most studies on the molecular genetics of the bladder cancer focus on changes in genomic DNA, including oncogenes and tumor suppressor genes, such as *HRAS, ERBB2, TP53* and *RB,* and subsequent cellular events [7,8].

Mitochondrial function and DNA attract less interest in studies on bladder carcinoma. Mitochondrial dysfunction has been linked to a wide range of degenerative and metabolic diseases, cancer, and even aging. mtDNA, which has a very high mutation rate, results in three classes of clinically relevant phenotypes: deleterious germline mtDNA mutations, which are linked to mitochondrial diseases; mtDNA polymorphisms, which are related to environmental adaptation in human evolution; and mtDNA somatic mutations, which are associated with aging and cancer. Mitochondrial defects were first associated with carcinogenesis several decades before, when Warburg reported “injury of the respiratory chain” and high glycolytic rate as typical of cancer [9-12].

Mitochondrial DNA is thought to accumulate more mutations than nuclear DNA (nDNA) to some extent, because the protective histones as well as the highly efficient DNA repair mechanisms do not exist in the mitochondrial nucleus. Certain tumors have been shown to result from mutations in nDNA-encoded mitochondrial proteins, which may result in increased reactive oxygen species (ROS) production. Mitochondrial dysfunction does appear to be a factor in cancer etiology. Alterations in mitochondrial DNA (mtDNA), including point mutations, deletions, insertions and genome copy number changes, are believed to be responsible for carcinogenesis [13-15]. For example, many reports have identified a mtDNA 4977-bp deletion in lung [16], breast [17] and endometrial carcinomas [18].

The use of mtDNA mutation and/or polymorphism patterns as a biomarker is rapidly expanding in disciplines, ranging from rare metabolic diseases and aging to cancer and the tracing of human migration patterns, population characterization and human identification in forensic science. In this study, we examined the presence of mutations in the mitochondrial D-Loop sequences of tumoral tissues as compared with adjacent non-tumoral tissues from Iranian patients with bladder cancer.

## Materials and Methods

Twenty-six men with primary urothelial bladder cancer with a mean age of 62.5 years were enrolled in this study (Table 1). The patients’ written consent was obtained and the institutional review board approved this study. Tumoral tissues were obtained from transurethral resection of the bladder tumor (TURBT) or radical cystectomy specimens. Tumoral tissues and adjacent non-tumoral tissues were immediately frozen in liquid nitrogen and kept at -80°C, while blood samples from patients were obtained before surgery.

**Table 1:** Age and histological type of primary urothelial bladder neoplasm sybtypes

**No. of Male patients**	**Age**	**Histological type**
1	62	Carcinoma in situ+
2	60	Papilloma
3	58	Papillary Urothelial carcinoma- low grade
4	63	Neoplasm of low malignant potential Papillary urothelial
5	73	Carcinoma in situ+
6	80	Papillary urothelial carcinoma – high grade
7	69	Papillary Urothelial carcinoma- low grade
8	68	Neoplasm of low malignant potential Papillary urothelial
9	53	Non-papillary urothelial carcinoma –high grade
10	55	Papillary urothelial carcinoma – high grade
11	75	Non-papillary urothelial carcinoma –high grade
12	78	Papillary Urothelial carcinoma- low grade
13	73	Neoplasm of low malignant potential Papillary urothelial
14	69	Papillary Urothelial carcinoma- low grade
15	68	Non-papillary urothelial carcinoma –high grade
16	57	Neoplasm of low malignant potential Papillary urothelial
17	53	Non-papillary urothelial carcinoma –high grade
18	50	Papillary urothelial carcinoma – high grade
19	49	Papillary Urothelial carcinoma- low grade
20	45	Non-papillary urothelial carcinoma –high grade
21	29	Papillary urothelial carcinoma – high grade
22	70	Papillary Urothelial carcinoma- low grade
23	66	Non-papillary urothelial carcinoma –high grade
24	58	Papillary urothelial carcinoma – high grade
25	59	Papillary Urothelial carcinoma- low grade
26	74	Neoplasm of low malignant potential Papillary urothelial

Urothelial bladder cancer diagnosis was done via histological analysis. Blood samples from healthy controls with a mean age of 57.5 years were obtained from 404 individuals of 17 ethnicities and 100 random individuals, all from the Tehran Special Medical Center. The exclusion criterion for the control group was any history of cancer, metabolic diseases and mitochondrial DNA related diseases that may affect the mtDNA. Ethics approval and patient informed consent including consent to participate in the study and consent to publish was obtained for the present study in accordance to the Tehran Special Medical Center and Medical Ethics Committee (Approval No. MS-16-2007).

### DNA extraction and sequencing

Genomic DNA (DNA fast, QIAGEN, Cat. No. 51204) was isolated from the tumoral tissues, adjacent non-tumoral tissues and blood samples of patients, as well as from the blood samples of controls, according to the manufacturer’s protocol. Two pairs of primers designed to amplify the mtDNA D-loop region are as follows: ONP 98 F )1579-15810(: 5′-ATC ATT GGA CAA GTA GCA TC -3′ and ONP 79R )780-761(: 5′-GAG CTG CAT TGC TGC GTG CT-3′. Polymerase chain reaction (PCR) was carried out with the following protocol: pre-denaturation at 95°C for 5 min, then 35 cycles of 94°C for 30 sec, 60°C for 45 sec and 72°C for 1 min, and a final extension step of 72°C for 6 min. Each amplified fragment was purified using a Agarose Gel DNA Fragment Recovery Kit, Ver.2.0 (TaKaRa, Japan) and subsequently sequenced using a ABI PRISM 3730 sequence analyzer (gene Fanavaran, Macrogene Seoul, Korea). The quality of the obtained chromatograms was assessed by FinchTV® software Version 1.4.0 (Geospiza, Inc., USA).

### Multiplex PCR

The PCR reactions were performed for 35 cycles of the following steps: 94°C for 10 min, 55°C for 10 min, and 72°C for 35 sec. Using the primers ONP 86, ONP 89, ONP 10, ONP 74, ONP 25 and ONP 99, the deletion-prone region between 5461 nt of the light strand and 15000 nt of the heavy strand was investigated in all the patients. The distances between the primers were long enough to allow amplification only if a part of the DNA between each respective primers was deleted. As a control in PCR analysis, a normal internal mtDNA fragment in a region which is seldom affected by deletions was amplified using the primer pair of ONP 86 and ONP 89 (Table 2). Polymerase chain reaction products were separated on 2% agarose gels and run in 0.5× Tris/Borate/EDTA buffer at 110 V for 50 min, stained in 0.002 μg/mL ethidium bromide, and visualized by means of an ultraviolet light.

**Table 2:** Primers used for detection of four deletions

**Forward Start point of primer**	**Reversed End point of primer**	**Length of deletion, kb**
ONP 86: 5461–5480	ONP 74: 15260–15241	8.7
5′-CCCTTACCACGCTACTCCTA -3′	5′-TGTCTACTGAGTAGCCTCCT-3′	
ONP 86: 5461–5480	ONP 10: 13640–13621	7.5
5′-CCCTTACCACGCTACTCCTA -3′	5′-GTTGACCTGTTAGGGTGAG-3′	
ONP 25: 8161–8180	ONP 10: 13640–13621	5
5′-CTACGGTCAATGCTCTGAAA-3′	5′-GTTGACCTGTTAGGGTGAG-3′	
ONP 25: 8161–8180	ONP 99: 16150–16131	7.5
5′-CTACGGTCAATGCTCTGAAA-3′	5′-GTGGTCAAGTATTTATGGTA-3′	
ONP 86: 5461–5480	ONP 89: 5740–5721	Internal Control
5′-CCCTTACCACGCTACTCCTA -3′	5′-GGCGGGAGAAGTAGATTGAA-3′	

### Southern blot analysis

Extracted mtDNA was eletrophoresed on 1% agarose gel. After electrophoresis, the DNA were denatured, neutralized and transferred to nylon membrane. Meanwhile, the ONP98 primer (5′-ATCATTGGACAAGTAGCATC-3′), located at 15791–15810 bp, and the ONP79 primer (5′-GAGCTGCATTGCTGCGTGCT-3′), located at 780–761 bp of the mtDNA, were used to amplify a 1558-bp fragment from the D-loop region. This fragment was used as a mtDNA probe. Southern blot analysis was performed using the DIG DNA Labeling and Detection Kit (Cat. #11093657910, Roche).

### Statistical analysis

Sequences were edited and aligned using ClustalX. The revised Cambridge Reference Sequence was used as a reference (GI: 251831106) (MITOMAP, 2009). The Chi-square test was used with SPSS (Statistical Package for the Social Sciences, version: 13) to examine the association of variations with control and patient samples. *P*-values < 0.05 were regarded as statistically significant.

## Results

**Figure 1:**
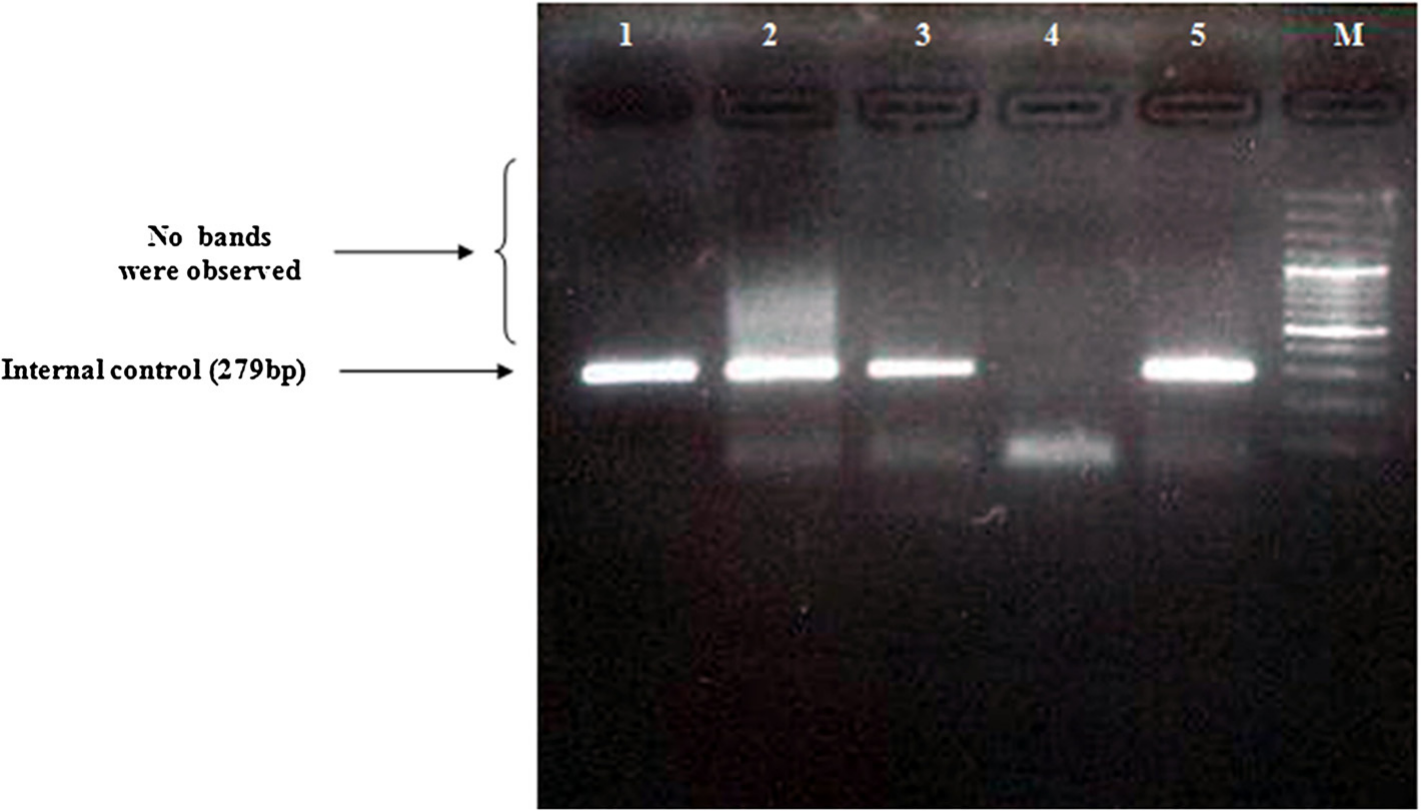
**Multiplex-PCR amplification.** Lanes 1, 2, 3 and 5 show the internal control (279 bp), lane 4 is the negative control and lane M is a 100 bp DNA size marker. No other bands were observed. Amplification only takes place if deletions occur in the DNA between the PCR primers.

**Figure 2:**
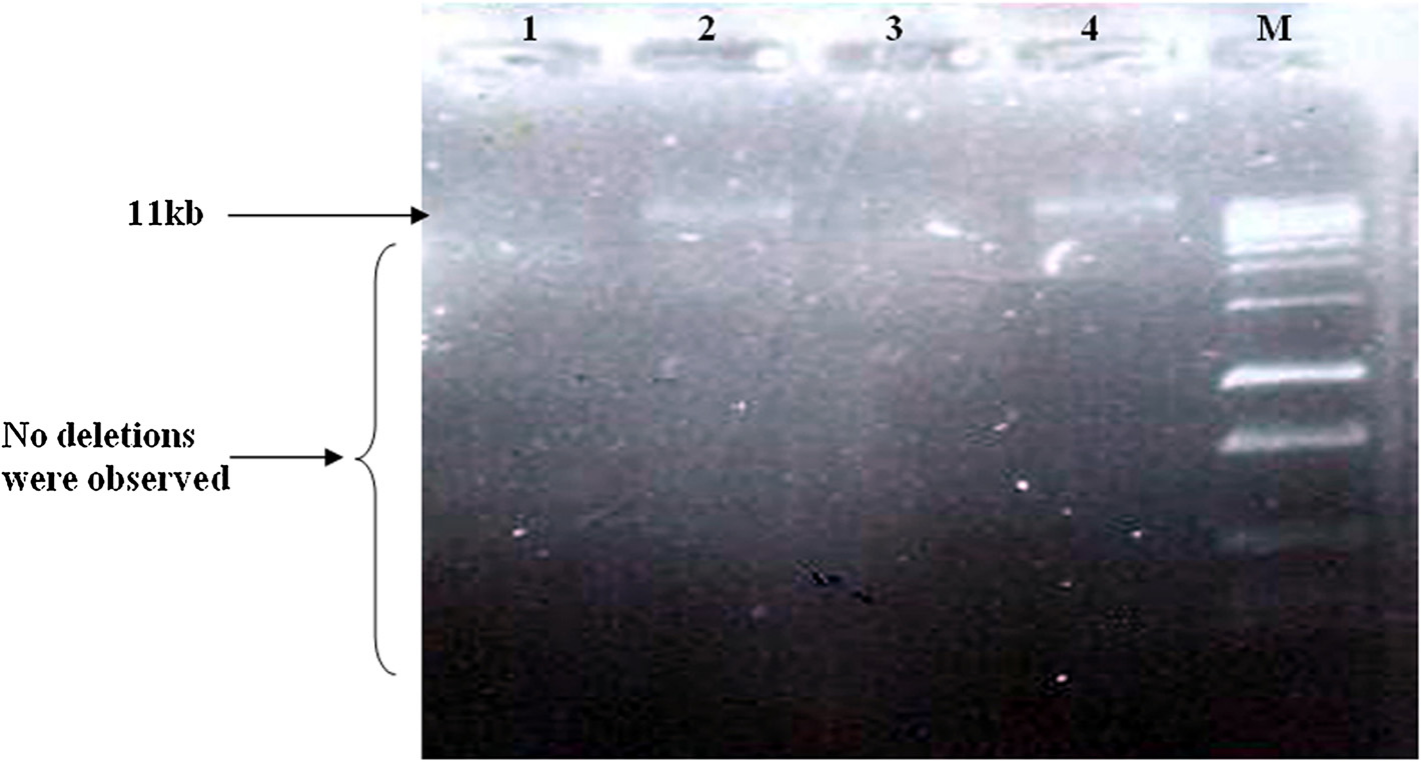
**Long range PCR amplification of mtDNA using Phusion Flash high-fidelity PCR Master Mix, Thermo Scientific.** A two-step long-range PCR was carried out on the major arc of the mitochondrial genome using the Expand Long Template PCR System to detect mitochondrial deletions. DNA products were separated using a 0.7% agarose gel containing ethidium bromide and viewed under UV light. Lanes 1 and 3: negative control; Lanes 2 and 4: an amplified 11 Kb fragment, indicating no deletions were observed in mtDNA; lane M: 1 kb DNA ladder marker.

**Figure 3:**
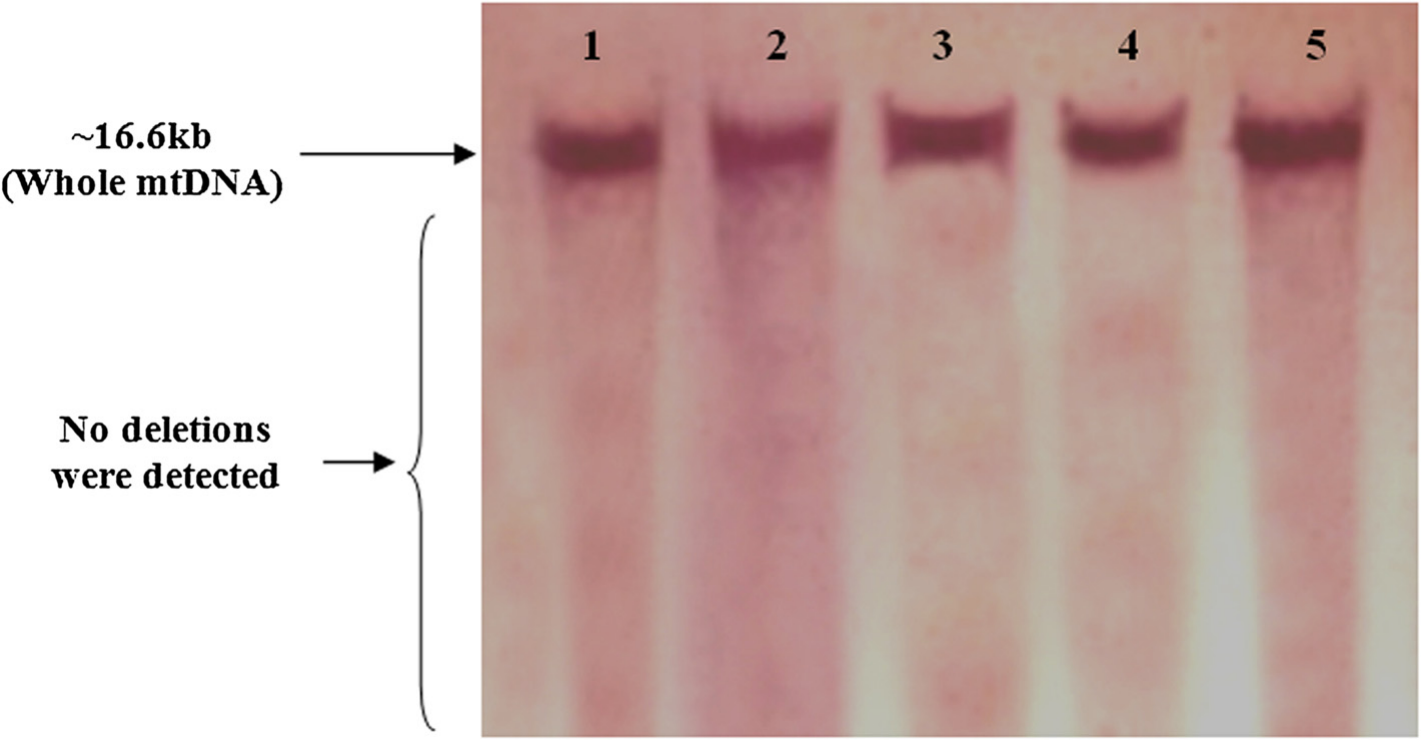
**Southern blot analysis of mitochondrial DNA (mtDNA) digested with the restriction enzyme BamH1 (nt14258), and hybridized with a DIG-labeled probe.** Lanes 1–5 shows intact mtDNA (~16.6 Kb).

Samples from a total of 26 patients with sporadic bladder cancer were screened for mitochondrial deletions and variations. Sequence analysis found a total of 110 variations (Cambridge Mitochondrial Sequences), of which 62 mutations were previously reported (MITOMAP). However, 48 of these mutations were reported as new mutations, which are summarized in Table 3. In this study, almost all of the variations were homoplasmic, but in 6 (16.6%) cases, a C nucleotide insertion was seen in locus 16194. No mitochondrial deletions were found in the patient samples (Figures 1 and 2), as confirmed by Southern blotting (Figure 3).

**Table 3:** List of variations in both healthy controls and bladder cancer patients

**NO.**	**Variations**	**Controls**	**Patients**
1	15968		
2	15969		
3	15996		
4	16004		
5	16017	*	
6	16021		
7	16026		
8	16033		*
9	16051		*
10	16067		*
11	16069		
12	16071		
13	16075		
14	16082		
15	16085		
16	16086		
17	16092		*
18	16093		
19	16095		
20	16111		*
21	16114		
22	16124		
23	16126		
24	16129	*	
25	16140		
26	16145		
27	16147	*	
28	16148		
29	16150	*	
30	16153		
31	16155		
32	16162		
33	16163		
34	16167		
35	16169		
36	16172		
37	16173		
38	16174	*	
39	16176	*	
40	16179		
41	16183		*
42	16184		
43	16187		*
44	16188		*
45	16189		
46	16192		
47	16193		
48	16201	*	
49	16203		
50	16207	*	
51	16209		
52	16213		
53	16217		*
54	16220		
55	16222		
56	16223		
57	16224		*
58	16227	*	
59	16230		*
60	16234		*
61	16239	*	
62	16242	*	
63	16243		
64	16245		
65	16247		*
66	16248		*
67	16249		
68	16256	*	
69	16261		
70	16263		
71	16264		
72	16265	*	
73	16266		
74	16270	*	
75	16274	*	
76	16278	*	
77	16286		
78	16287	*	
79	16288		
80	16290		*
81	16292		
82	16294		
83	16295		
84	16296		
85	16298	*	
86	16304	*	
87	16309		
88	16311		
89	16318		*
90	16318		
91	16319		
92	16320		*
93	16324	*	
94	16325	*	
95	16327		*
96	16342		
97	16343		
98	16352	*	
99	16354		
100	16355	*	
101	16356		*
102	16362		
103	16390	*	
104	16391	*	
105	16399	*	
106	16413		
107	16468		
108	16482	*	
109	16497	*	
110	16527		*

*Indicates novel mutation has not been reported before.

**Figure 4:**
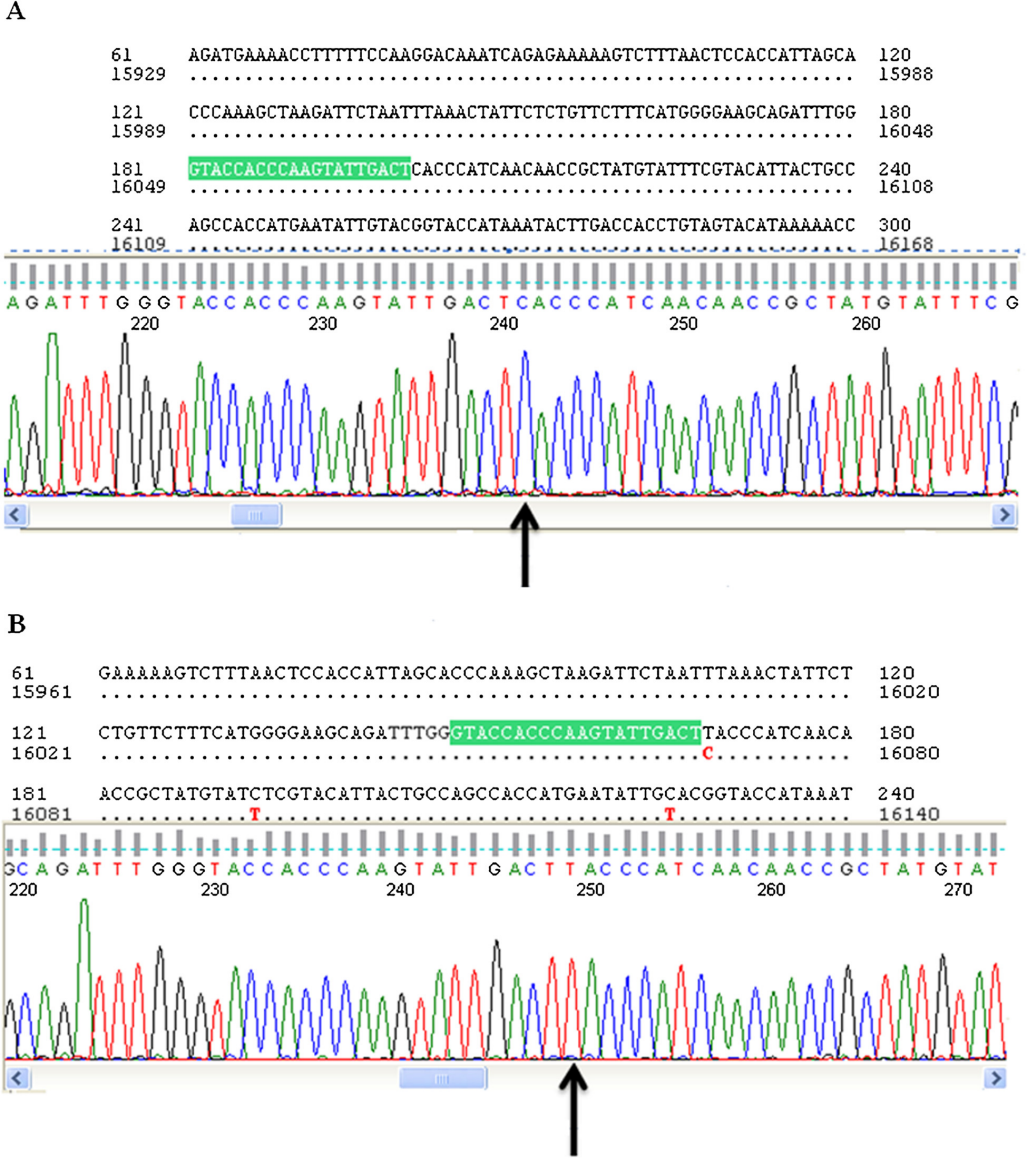
**Chromatogram showing homoplasmy at position 16069 of the mitochondrial DNA D-loop in a normal sequence (Figure**4**-A) and a variation (Figure**4**-B).** The arrow marks the sequence variations.

Four common variations, 16069, 16189, 16261 and 16311, were found in the tumoral tissues, adjacent non-tumoral tissues and blood samples of both patients and controls from different ethnicities. The polymorphisms at 16189, 16261 and 16311 were not significantly correlated with bladder cancer. However, the D-loop C16069T polymorphism (Figure 4) was significantly correlated with bladder cancer (*P* < 0.05). Analysis of control samples by ethnicities for these 4 variations is summarized in Table 4. No significant difference (*p* > 0.05) in D310 C variations was observed between the patient and control samples (Table 5).

**Table 4:** Comparison of 4 common variations in bladder cancer patients and controls

**Ethnicity**	**NO.**	**16069**	**16189**	**16261**	**16311**
**Arab**	23	0 (0%)	7 (30.4%)	4 (17.4%)	4 (17.4%)
**Armenian**	18	0 (0%)	5 (27.7%)	2 (11%)	3 (16.7%)
**Asurian**	19	1 (5.2%)	7 (36.8%)	3 (15.8%)	1 (5.3%)
**Azari**	22	0 (0%)	6 (27.3%)	0 (0%)	4 (18.2%)
**Turkmen**	37	1 (2.7%)	5 (13.5%)	1(2.7%)	6 (16.2%)
**Baluch**	13	0 (0%)	2 (15.4%)	1 (7.7%)	0 (0%)
**Bandari**	31	0 (0%)	13 (42%)	2 (6.5%)	5 (13.5%)
**Guilani**	24	0 (0%)	2 (8.3%)	3 (12.5%)	3 (12.5%)
**Jews**	37	1 (2.7%)	6 (16.2%)	4 (10.8%)	4 (10.8%)
**Kurd**	24	2 (8.3%)	3 (12.5%)	4 (16.7%)	6 (25%)
**Lur**	22	0 (0%)	1 (4.5%)	8 (36.4%)	8 (36.4%)
**Mazani**	23	0 (0%)	4 (17.4%)	4 (17.4%)	1 (4.3%)
**Persian Isfahan**	16	0 (0%)	6 (37.5%)	2 (12.5%)	5 (31.2%)
**Persian Kerman**	25	0 (0%)	7 (28%)	1 (4%)	5 (20%)
**Persian Mashhad**	23	0 (0%)	5 (21.7%)	2 (8.7%)	2 (8.7%)
**Persian Shiraz**	23	0 (0%)	4 (17.4%)	2 (8.7%)	3 (3.2%)
**Persian Yazd**	24	0 (0%)	5 (20.8%)	1 (4.1%)	2 (8.3%)
**Mixed Tehran**	100	8 (8%)	9 (9%)	9 (9%)	12 (12%)
**Total (controls)**	504	13 (2.6%)	95 (18.8%)	53 (10.5%)	78 (15.5%)
**Patients**	26	5 (19%)*	4 (15.4%)	4 (15.4%)	8 (31%)

*Shows statistically significant, *p* < 0.05.

**Table 5:** Association of the mtDNA D310 variation in bladder cancer patients and controls

**Ethnicity**	**NO.**	**C 7TC6**	**C 8TC6**	**C 9TC6**	**C 10TC6**
**Arab**	23	12 (52.2%)	8 (34.7%)	2 (8.7%)	1 (4.3%)
**Armenian**	18	7 (38.9%)	11 (61.1%)	0 (0%)	0 (0%)
**Azari**	22	8 (36.4%)	12 (54.5%)	2 (9%)	0 (0%)
**Turkmen**	37	17 (45.9%)	16 (43%)	4 (10.8%)	0 (0%)
**Bandari**	31	10 (32%)	15 (48.4%)	6 (19.4%)	0 (0%)
**Persian Isfahan**	16	5 (31.3%)	9 (56.3%)	2 (6.5%)	0 (0%)
**Persian Mashhad**	23	14 (60.9%)	8 (34.8%)	1 (4.3%)	0 (0%)
**Persian Shiraz**	23	6 (26%)	16 (69.6)	1 (4.3%)	0 (0%)
**Persian Yazd**	24	9 (37.5%)	9 (37.5%)	5 (20.8%)	1 (4.1%)
**Guilani**	24	8 (33%)	12 (50%)	4 (16.6%)	0 (0%)
**Jews**	37	16 (43%)	17 (45.9)	4 (10.8%)	0 (0%)
**Kurd**	24	3 (12.5%)	14 (58%)	7 (29%)	0 (0%)
**Lur**	22	9 (41%)	9 (41%)	4 (18%)	0 (0%)
**Total (controls)**	324	124 (38.3)	156 (48.1%)	42 (13%)	2 (0.6%)
**Patients**	21	9 (42.9%)	10 (47.6%)	2 (9.5%)	0 (0%)

The D310 sequence variations of mtDNA in patients and controls were not significantly different (*p* > 0.05).

## Discussion

Our sequencing analysis focused on the mtDNA D-loop region, which is highly polymorphic and contains two hypervariable regions, HV1 (16024–16383) and HV2 (57–333), that was considered as a somatic mutation “hot spot” in many types of cancer [19]. In this study, no deletions were seen in the mitochondrial genome. One hundred and sixteen variations were observed in the D-Loop region, where 48 of them were not previously reported. Wada *et al.*[20] also reported that the majority of somatic mutations were homoplasmic, suggesting that the mutant mtDNA became dominant in tumor cells. Fliss *et al.*[21] screened 14 urinary bladder cancers for somatic mutations in the D-loop region, and found mutations in 4 (29%) samples.

Polymorphism 16189, which is highly polymorphic, was the previous focus of oncological research because carriers with the T16189C polymorphism were apparently more susceptible to breast cancer and ganglioma development. Interestingly, the T16189C polymorphism was found in 14% of endometrial cancers [22] and type II diabetes mellitus [23,24].

In this study, in contrast to 16189, 16194, 16261 and 16311 variations, the C16069T polymorphism of the D-loop indicated significant correlation with bladder cancer (P < 0.05), which has not been studied in bladder cancer before. However, the C16069T polymorphism has been reported in prostate cancer [25], pancreatic cancer [26], endometrial cancer [27], breast cancer [28,29], repeated pregnancy loss [30] and age-related macular degeneration [31]. This result supports our hypothesis, which shows the potential of specific mitochondrial 16069 polymorphism involvement in carcinogenesis.

Many studies reported that the C150T polymorphism is correlated with longevity (MITOMAP, 2009). The possible function of the C150T transition was investigated in a previous study [32], suggesting that the C150T transition functions in remodeling mtDNA replication. However, in our study, no significant differences were found between C150T mutations in patients and control samples from different ethnicities.

Large-scale mtDNA deletions have been demonstrated in several cancers. Kamalidehghan *et al.*[33] found that the common mtDNA4977 deletion was less frequent in gastric cancer tissues compared to the normal adjacent tissues. While in another study, a deletion of approximately 8.9 kb was more frequent in gastric carcinoma tissues than adjacent normal tissue samples [34]. However, in the present study, no deletions were detected in bladder carcinoma tissues nor adjacent non-tumoral tissues. Therefore, the pattern of mitochondrial deletions may differ among different carcinomas.

Marchington *et al.*[35] first used the term D310 to describe a highly polymorphic mononucleotide tract of poly (C) that varies from 12 to 18 Cs, located between nucleotide positions 303 and 318 in CSB II, that forms a RNA–DNA hybrid known as an R-loop. This poly(C) region is interrupted at nucleotide position 310 by a T (CCCCCCCTCCCCC), in which the number of Cs before the T can vary between 7 to 9 in normal polymorphic variants [35]. D310 has been reported as a mutational hot-spot in a large panel of tumors including gastric, head and neck, breast, colorectal, lung and bladder cancers, where head and neck cancer has the highest rate of D310 variants (37%), followed by breast (29%) and colorectal (28%) cancers. However, no D310 alterations were detected in prostate and ovarian cancers [36,37].

The D310 region of mtDNA plays an important role in mitochondrial biogenesis, where somatic insertions or deletions of one or two base pairs in this region are thought to have negligible effects on cancers. However, major deletions or insertions of up to ten bases in the D310 region could interfere with mtDNA biogenesis [38]. Mutations in the D-loop, mostly at D310, have been found in 21% of all head and neck squamous cell carcinomas [39]. However, in our study, the D310 mtDNA sequence variations, including C7TC6, C8TC6, C9TC6 and C10TC6, were not significantly different (p > 0.05) between bladder cancer patients and controls of different ethnicities.

In conclusion, our study suggests that the mitochondrial DNA D-Loop 16069 mutation may play a significant role in the etiology of bladder cancer and facilitate the definition of carcinogenesis-related mutations in human mtDNA.

## Abbreviations

mtDNA: mitochondrial DNA; D-Loop: Displacement loop; PCR: Polymerase chain reaction; nDNA: nuclear DNA; ROS: Reactive oxygen species; TURBT: Transurethral resection of the bladder tumor; SPSS: Statistical package for the social sciences; HV: Hypervariable; mtMSI: Mitochondrial microsatellite instability.

## Authors’ contributions

NS, AF, SD, MM and RS carried out the experimental procedures. BK and RR wrote and edited the manuscript and performed the statistical analysis. MH conceived the project and supervised the study. All authors read and approved the final manuscript.

## Competing of interests

The authors declare that they have no competing of interests.
